# A new WHO bottle bioassay method to assess the susceptibility of mosquito vectors to public health insecticides: results from a WHO-coordinated multi-centre study

**DOI:** 10.1186/s13071-022-05554-7

**Published:** 2023-01-20

**Authors:** Vincent Corbel, Mara D. Kont, Martha Liliana Ahumada, Laura Andréo, Bazoma Bayili, Koama Bayili, Basil Brooke, Jesús A. Pinto Caballero, Ben Lambert, Thomas S. Churcher, Stephane Duchon, Josiane Etang, Adriana E. Flores, Kasinathan Gunasekaran, Waraporn Juntarajumnong, Matt Kirby, Rachel Davies, Rosemary Susan Lees, Audrey Lenhart, José Bento Pereira Lima, Ademir J. Martins, Pie Müller, Raphael N’Guessan, Corine Ngufor, Giorgio Praulins, Martha Quinones, Kamaraju Raghavendra, Vaishali Verma, Adanan Che Rus, Michael Samuel, Koou Sin Ying, Sungsit Sungvornyothin, Sreehari Uragayala, Raman Velayudhan, Rajpal S. Yadav

**Affiliations:** 1 Institut de Recherche pour le Développement (IRD), MIVEGEC, CNRS, IRD, Université de Montpellier, 911 Av Agropolis, 34 394 Montpellier, France; 2 MRC Centre for Global Infectious Disease Analysis, Department of Infectious Disease Epidemiology, Imperial College London (ICL), Norfolk Place, London, W2 1PG UK; 3 Grupo de Entomología, Instituto Nacional de Salud, Avenida calle 26 No. 51-20—Zona 6 CAN, 111321 Bogotá D.C., Colombia; 4 Institut de Recherche en Sciences de la Santé (IRSS), 399 Avenue de la liberte., 01 BP 545, Bobo-Dioulasso 01, Burkina Faso; 5 Centre for Emerging Zoonotic and Parasitic Diseases, National Institute for Communicable Diseases/NHLS and Wits Research Institute for Malaria, Faculty of Health Sciences, University of the Witwatersrand, Johannesburg, South Africa; 6 Laboratorio de Referencia Nacional de Entomología (LRNE), Centro Nacional de Salud Pública, Instituto Nacional de Salud, Av. Defensores del Morro 2268 (Ex Huaylas) Chorrillos, Lima 9-(511) 748-0000, Anexo 1548, Lima, Peru; 7 Department of Mathematics, University of Exeter, North Park Rd, Exeter, EX4 4QF UK; 8 Laboratoire de Recherche sur le Paludisme, Institut de Recherche de Yaoundé (IRY)–Organisation de Coordination pour la lutte contre les Endémies en Afrique Centrale (OCEAC), BP 288, Yaoundé, Cameroun; 9 Department of Biological Sciences, Faculty of Medicine and Pharmaceutical Sciences, University of Douala, P.O. Box 2701, Douala, Cameroon; 10 Facultad de Ciencias Biologicas, Laboratorio de Entomologia Medica, Universidad Autónoma de Nuevo León (UANL), Av. Universidad S/N Ciudad Universitaria, 66455 San Nicolas de los Garza, NL Mexico; 11 Indian Council of Medical Research-Vector Control Research Centre (VCRC), Indira Nagar, Puducherry, 605006 India; 12 Department of Entomology, Faculty of Agriculture, Kasetsart University (KU), 50 Ngam Wong Wan Rd., Lat Yao, Chatuchak, Bangkok, 10900 Thailand; 13 Kilimanjaro Christian Medical Centre (KCMC), Kilimanjaro Christian Medical University College–The Pan African Malaria Vector Research Consortium (KCMUCo-PAMVERC), Off Sokoine Road, PO Box 2228, Moshi, Kilimanjaro Tanzania; 14 London School of Hygiene and Tropical Medicine (LSHTM), Keppel Street, London, WC1E 7HT UK; 15 Vector Biology Department, Liverpool School of Tropical Medicine (LSTM), Pembroke Place, Liverpool, L3 5QA UK; 16 Entomology Branch, Centers for Disease Control and Prevention (CDC), 1600 Clifton Rd, Atlanta, GA 30329 USA; 17 Laboratório de Fisiologia E Controle de Artrópodes Vetores (Laficave), Instituto Oswaldo Cruz (IOC), Fundacao Oswaldo Cruz (FIOCRUZ), Avenida Brasil, 4365 Manguinhos, Rio de Janeiro, RJ 21040-360 Brazil; 18 Swiss Tropical and Public Health Institute (Swiss TPH), Kreuzstrasse 2, 4312 Allschwil, Switzerland; 19 University of Basel, Petersplatz 1, PO Box 4001, Basel, Switzerland; 20 Face Ecole des Infirmiers, Institut Pierre Richet (IPR), Institut National de Santé Publique (INSP), 01 BP 1500 Bouaké, Côte d’Ivoire; 21 Centres de Recherches Entomologiques de Cotonou (CREC), Ministère de la santé du Bénin, BP 2604, Cotonou, Benin; 22 Public Health Department, Faculty of Medicine, Universidad Nacional de Colombia, Bogota, Colombia; 23 Indian Council of Medical Research-National Institute of Malaria Research (NIMR), Sector-8, Dwarka, New Delhi 110077 India; 24 Vector Control Research Unit (VCRU), School of Biological Sciences, Universiti Sains Malaysia, Penang, Malaysia; 25 Environmental Health Institute (EHI), National Environmental Agency (NEA), 11 Biopolis Way, #06-05/08 Helios Block, Singapore, 138667 Singapore; 26 Department of Medical Entomology, Faculty of Tropical Medicine, Mahidol University (MU), 420/6 Ratchawithi Road, Ratchathewi, Bangkok, 10400 Thailand; 27 Field Unit, Indian Council of Medical Research (ICMR)-National Institute of Malaria Research (NIMR), Poojanahalli, Kannamangla Post, Devanahalli Taluk, Bengaluru, 562110 India; 28 Vector Control, Veterinary Public Health and Environment Unit, Department of Control of Neglected Tropical Diseases, World Health Organization, 20 Avenue Appia, 1211 Geneva 27, Switzerland

**Keywords:** *Aedes*, *Anopheles*, Insecticide resistance, Public health insecticides, Susceptibility test, Vector control, WHO bottle bioassay, Discriminating concentrations

## Abstract

**Background:**

The continued spread of insecticide resistance in mosquito vectors of malaria and arboviral diseases may lead to operational failure of insecticide-based interventions if resistance is not monitored and managed efficiently. This study aimed to develop and validate a new WHO glass bottle bioassay method as an alternative to the WHO standard insecticide tube test to monitor mosquito susceptibility to new public health insecticides with particular modes of action, physical properties or both.

**Methods:**

A multi-centre study involving 21 laboratories worldwide generated data on the susceptibility of seven mosquito species (*Aedes aegypti*, *Aedes albopictus*, *Anopheles gambiae* sensu stricto [*An. gambiae* s.s.], *Anopheles funestus, Anopheles stephensi, Anopheles minimus* and *Anopheles albimanus*) to seven public health insecticides in five classes, including pyrethroids (metofluthrin, prallethrin and transfluthrin), neonicotinoids (clothianidin), pyrroles (chlorfenapyr), juvenile hormone mimics (pyriproxyfen) and butenolides (flupyradifurone), in glass bottle assays. The data were analysed using a Bayesian binomial model to determine the concentration–response curves for each insecticide–species combination and to assess the within-bioassay variability in the susceptibility endpoints, namely the concentration that kills 50% and 99% of the test population (LC_50_ and LC_99_, respectively) and the concentration that inhibits oviposition of the test population by 50% and 99% (OI_50_ and OI_99_), to measure mortality and the sterilizing effect, respectively.

**Results:**

Overall, about 200,000 mosquitoes were tested with the new bottle bioassay, and LC_50_/LC_99_ or OI_50_/OI_99_ values were determined for all insecticides. Variation was seen between laboratories in estimates for some mosquito species–insecticide combinations, while other test results were consistent. The variation was generally greater with transfluthrin and flupyradifurone than with the other compounds tested, especially against *Anopheles* species. Overall, the mean within-bioassay variability in mortality and oviposition inhibition were < 10% for most mosquito species-insecticide combinations.

**Conclusion:**

Our findings, based on the largest susceptibility dataset ever produced on mosquitoes, showed that the new WHO bottle bioassay is adequate for evaluating mosquito susceptibility to new and promising public health insecticides currently deployed for vector control. The datasets presented in this study have been used recently by the WHO to establish 17 new insecticide discriminating concentrations (DCs) for either *Aedes* spp. or *Anopheles* spp. The bottle bioassay and DCs can now be widely used to monitor baseline insecticide susceptibility of wild populations of vectors of malaria and *Aedes-*borne diseases worldwide.

**Graphical abstract:**

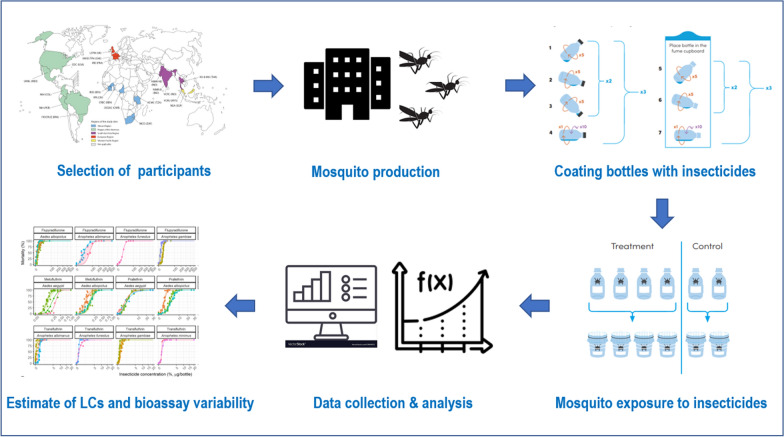

**Supplementary Information:**

The online version contains supplementary material available at 10.1186/s13071-022-05554-7.

## Background

Over 80% of the world’s population lives in regions at risk of at least one vector-borne disease [1]. Insecticide-based vector control interventions play a pivotal role in vector control programmes for the prevention, control and elimination of vector-borne diseases by interrupting transmission and reducing morbidity and mortality in various disease transmission settings [2]. Insecticide resistance of mosquitoes is expanding both in frequency and intensity, thus causing an increasing threat to the control and prevention of mosquito-borne diseases [3, 4]. According to the latest reports, *Anopheles* spp. vectors resistant to insecticides from at least one mode of action class were found in 78 countries with ongoing malaria transmission, with resistance to the pyrethroids being the most common [5]. A systematic review [6] reported the presence of insecticide resistance in *Aedes* spp. in at least 57 countries, with evidence for strong resistance in Asia and South America [7]. The continued spread of insecticide resistance in mosquitoes threatens to undermine progress made thus far in controlling malaria and arboviral diseases and may lead to operational failure of insecticide-based control measures [3, 8] if resistance management is not implemented at an operational scale.

The Global Vector Control Response 2017–2030 (GVCR) adopted by the World Health Assembly in May 2017 has put vector surveillance and control back on the public health agenda as a strategic approach to reduce the burden and threat of vector-borne diseases that affect human populations [9]. As large amounts of insecticides are used globally for the control of vector-borne diseases (i.e. > 5000 metric tonnes of active ingredients annually between 2010 and 2019 [10]), insecticide resistance monitoring is critical to enhance vector surveillance and monitoring and the evaluation of interventions. It can help generate the required evidence on the status of insecticide resistance in mosquito populations to guide vector-control programmes on the selection and deployment of efficacious insecticidal products for vector control and insecticide resistance management [11]. Given the evidence of increasing resistance among malaria and dengue vectors to the insecticides in the four main mode of action classes deployed (i.e. organochlorines, pyrethroids, organophosphates and carbamates) and the potential risk for cross-resistance to new public health insecticides, attention has focussed on the need for more intensive and improved monitoring of insecticide resistance in mosquitoes.

Insecticide resistance in field populations of mosquitoes is monitored by measuring mosquito mortality in tube test bioassays in which mosquitoes are exposed to filter papers impregnated with a diagnostic or discriminating concentration (DC) of insecticide. The WHO has traditionally defined insecticide DCs as either twice the lowest concentration that systematically gives 100% mortality (i.e. lethal concentration [LC] 100 or LC_100_) or twice the estimated LC that gives 99.9% mortality (LC_99.9_) of a susceptible mosquito colony scored 24 h after a 1-h exposure time [12]. The concept of a DC has clear benefits in terms of the cost and the efficiency of testing and has been adopted to monitor insecticide resistance of mosquitoes and other vectors of diseases [13, 14]. Currently, insecticide-impregnated papers are produced for monitoring the resistance of *Anopheles*, *Aedes* and *Culex* spp., with the DCs applied having been determined in a number of historical studies prior to 1998 as well as from the results of a 1998 WHO study with *Anopheles* spp. vectors [12]. Tentative DCs of some new insecticides were subsequently adopted by WHO for testing resistance in *Anopheles* spp. as an interim measure [13]. However, since 1998 no other DCs have been validated by WHO for *Anopheles* spp. and/or *Aedes* spp. [14], either for insecticides already in public health use (e.g. some pyrethroids) or new ones with novel modes of action (e.g. pyrroles, juvenile hormone mimics, neonicotinoids and butenolides) that have been prequalified by WHO or are under evaluation for vector control.

Recently, some attempts to establish DCs for two new and promising insecticides, the neonicotinoid clothianidin and the pyrrole chlorfenapyr, did not succeed due to their instability when impregnated on filter papers which limited the bio-efficacy testing and shelf-life of the treated papers [15–18]. The US Centers for Disease Control and Prevention (CDC) developed a bottle bioassay as an alternative to the WHO tube test that also lends itself to the assessment of mosquito resistance to insecticides that were found to be unsuitable for impregnating filter papers [19]. The bottle bioassay offers more flexibility and practicability compared to insecticide-impregnated papers and allows for the use of additives or surfactants that can prevent the crystallization of the insecticide compounds, ensuring uniform coating of the bottles [16, 20]. The suitability and practicability of glass bottles for monitoring resistance in mosquitoes has led to recent proposals for DCs for clothianidin and chlorfenapyr [15–17, 20], albeit using a different study design and test conditions that make interpretation of the results extremely difficult. Furthermore, the CDC bottle bioassay provides an estimation of the length of time to knockdown or incapacitation of mosquitoes within a 2-h exposure time while mosquito mortality 24 h after a 1-h exposure is the endpoint advocated by the WHO for monitoring resistance in the WHO tube test method. A comparative study showed that time-to-knockdown in the CDC bottle bioassay is a poor predictor of 24 h mortality in the tube test [21] and, therefore, data originating from the two bioassays cannot be directly compared.

As new compounds with novel modes of action are discovered or repurposed from agriculture to public health use, it was considered essential to develop and standardize a WHO bottle bioassay method to monitor mosquito susceptibility to insecticides using endpoints similar to those of the current WHO tube test. Such an assay would allow for monitoring baseline susceptibility of malaria and dengue vectors in the field to new compounds to guide at-risk countries for the deployment of new tools, replacing ineffective insecticide-based vector control tools.

A WHO-sponsored and coordinated multi-centre study involving 21 laboratories worldwide was conducted from 2017 to 2021 to develop standard operating procedures and to determine the DCs of 18 insecticides in five *Anopheles* and two *Aedes* species [22]. Participating laboratories were selected after a WHO consultation with key stakeholders, and standardized test protocols were developed to generate susceptibility data with well-characterized susceptible mosquito colonies. The present paper reports the bioassay test results obtained for seven of the 18 compounds that were selected for testing in the newly developed glass bottle bioassay method, which was adapted from the CDC protocol. A Bayesian binomial model was developed to analyse the large datasets coming from the multi-centre study in order to determine concentration–response estimates and to assess variability in mortality under different test conditions. Overall the results obtained validate the bottle method to produce the data needed to assess mosquito susceptibility to insecticides and establish DCs for compounds which are not suitable for use on filter papers. This method can be used, with the DCs recently established [22], to monitor for resistance to these compounds in wild mosquito populations.

## Methods

### Study sites

**Fig. 1:**
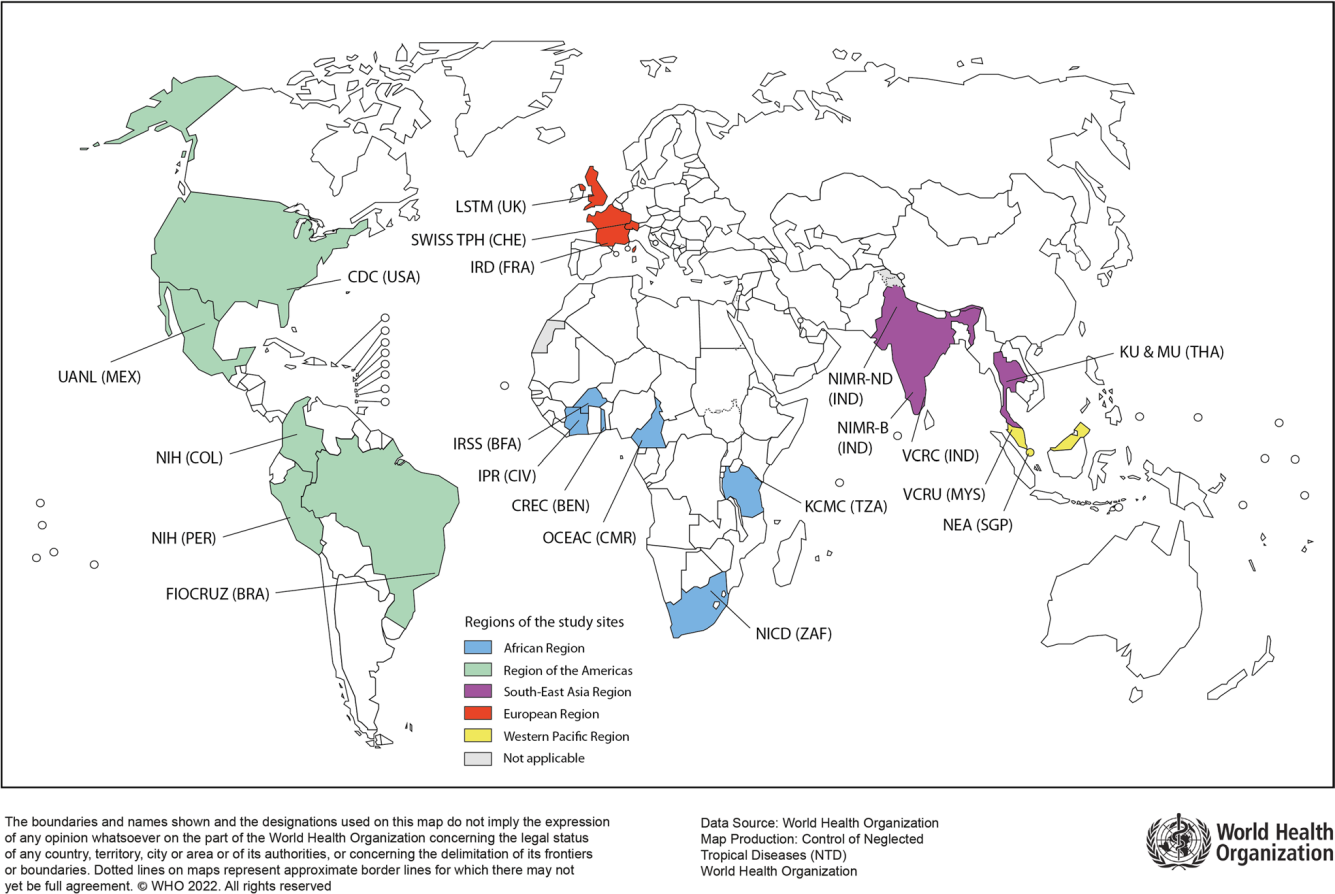
Location of participating laboratories by WHO Region. European Region: Institut de Recherche pour le Développement (IRD), France; Swiss Tropical and Public Health Institute (Swiss TPH), Switzerland; Liverpool School of Tropical Medicine (LSTM), UK. African Region: Institut Pierre Richet (IPR), Cote d’Ivoire; Centres de Recherches Entomologiques de Cotonou (CREC), Benin; Institut de Recherche en Sciences de la Santé (IRSS), Burkina Faso; Organisation de coordination et de coopération pour la lutte contre les grandes endémies en Afrique Centrale (OCEAC), Cameroon; National Institute for Communicable Diseases (NICD), South Africa; Kilimanjaro Christian Medical University College (KCMC), Tanzania. South-East Asian Region: ICMR-Vector Control Research Centre (VCRC), India; ICMR-National Institute of Malaria Research, New Delhi (NIMR-D); Bengaluru (NIMR-B), India; Kasetsart University (KU), Thailand; Mahidol University (MU), Thailand. Western-Pacific Region: Vector Control Research Unit (VCRU), Malaysia; National Environmental Agency (NEA), Singapore; American Region: Fundacao Oswaldo Cruz (FIOCRUZ), Brazil; National Institute of Health (NIH), Colombia; National Institute of Health (NIH), Peru; Universidad Autónoma de Nuevo León (UANL), Mexico; Centers for Disease Prevention and Control (CDC), USA

Overall, 21 internationally recognized laboratories from five WHO regions participated in the study, which was jointly coordinated by Institut de Recherche pour le Développement (IRD) and the WHO (Fig. 1). These 21 laboratories were selected because they were either formally designated WHO Collaborating Centres or had adequate facilities and the capacity for conducting bioassays according to the WHO standards. All laboratories maintained mosquito colonies that were reported to be susceptible to the insecticides under testing in this study.

### Mosquito species and strains

Two major arbovirus vector species, *Aedes aegypti* and *Aedes albopictus*, and five major malaria vector species, *Anopheles albimanus*,* Anopheles gambiae* sensu stricto (*An. gambiae* s.s.),* Anopheles funestus* sensu stricto (*An. funestus* s.s.), *Anopheles minimus* sensu stricto (*An. minimus* s.s.) and *Anopheles stephensi*, were included in the study. These species were selected because they play a primary role in the transmission of malaria, dengue, chikungunya and/or Zika viruses in different geographical regions of Africa, Asia-Pacific, Central and South America and the Middle East, and each was held in colony by at least three different participating laboratories to enable cross-validation tests to be conducted at multiple sites. Different colonies of each species were used by the participating laboratories for testing insecticides (Table 1). The mosquitoes had all been a colony for several generations, and each colony had previously been shown to be susceptible to the tested insecticides using biological and/or molecular assays.

**Table 1:** Mosquito species and strains selected for the WHO multi-centre laboratory study

Species	Strains
*Aedes aegypti* (Linnaeus, 1762)	Bora Bora, New Orleans, Rockefeller
*Aedes albopictus* (Skuse, 1895)	Chengdu*,* EHI, NEA, Perols, VCRU
*Anopheles gambiae* sensu stricto (Giles, 1902)	Kisumu
*Anopheles funestus* sensu stricto (Giles, 1900)	Fang
*Anopheles stephensi* (Liston, 1901)	NDD, Puducherry
*Anopheles minimus* sensu stricto (Theobald, 1901)	TM
*Anopheles albimanus* (Wiedemann, 1820)	Sanarate, Buenaventura

### Insecticides

Following a WHO consultation in 2017 with experts and researchers from academia and industry, 18 insecticides were determined to be of high priority for testing against *Anopheles* and/or *Aedes* species [22]. Seven of these insecticides (see Table 2), belonging to five different mode of action classes, were tested in glass bottles because they were not suitable for impregnation onto filter papers due to the instability of the treated papers. These compounds are used in various formulations of insecticidal products that are currently in use or were under WHO evaluation for vector control, including for use in indoor residual sprays or in insecticide-treated nets, space sprays, household pesticide products and/or spatial repellents.

**Table 2:** Insecticides successfully tested with WHO bottle bioassays

Class	Insecticide	CAS RN^a^	Test mosquitoes		Product type^b^
			*Anopheles* spp.	*Aedes* spp.	
Pyrroles	Chlorfenapyr	122453-73-0		−	ITN
Neonicotinoids	Clothianidin	210880-92-5		tested	IRS
Juvenile hormone mimics	Pyriproxyfen	95737-68-1	tested	−	ITN/Larvicide
Butenolides	Flupyradifurone	951659-40-8	tested	tested	SP
Pyrethroids	Transfluthrin	118712-89-3	tested	tested	SS
	Prallethrin	23031-36-9	−	tested	SS
	Metofluthrin	240494-71-7	−	tested	SR

IRS Indoor residual spraying, ITN insecticide-treated nets, SS space spray, SR spatial repellents

^a^Chemical Abstract Service Registry Number

^b^Primary method of application for a given insecticide

### Study design

The first objective of the study was to develop and cross-validate protocols for testing the selected insecticides in a glass bottle bioassay. Six laboratories with strong expertise in testing and evaluating public health pesticides were selected by the WHO to determine the most suitable technical parameters for applying the WHO bottle bioassay using each insecticide. These parameters included the coating of bottles, the bottle drying time (i.e. 1, 2 or 24 h), the mosquito exposure time (1 or 2 h), the post-exposure holding period (24, 48 or 72 h) and appropriate test conditions, including ambient temperature, relative humidity and optimum concentrations of MERO®, a surfactant made of 81% rapeseed oil methyl ester that was supplied by Bayer CropScience (Mohnheim, Germany). The test protocols were validated in subsequent WHO consultations once results were consistent and reproducible across three independent laboratories for a given insecticide (see details in [22]).

After validating the test protocols, we conducted a multi-centre study with serial concentrations of the insecticides to generate concentration–response data for each insecticide–species combination. For insecticides with a killing action, the first step of the study (Step 1) comprised preliminary bioassays to scope the range of insecticide concentrations that would kill between 0 and 100% of the tested mosquito colonies using a small number of mosquitoes (*n* = 50 per concentration). Based on Step 1, a range of six insecticide concentrations were chosen to produce a complete set of bioassays in triplicate (Step 2) (*n* = 300 per concentration). Based on the results, we estimated the lethal concentrations that would kill 50% and 99% (LC_50_ and LC_99_) of the tested mosquito colonies using a statistical model specifically developed for analysing complex toxicological datasets (see details in section Statistical analysis). For pyriproxyfen, which inhibits or reduces the fertility and fecundity of adult female mosquitoes, we estimated the concentrations that inhibit oviposition by 50% and 99% (oviposition inhibition (OI)_50_ and OI_99_ respectively) by the end of the observation period.

### WHO bottle bioassay procedure

#### Preparation of stock solutions

The following insecticides were tested using high-purity technical-grade active ingredients: (i) transfluthrin (99.2%), flupyradifurone (98.4%) and clothianidin (99.4%), from Bayer CropScience (Mohnheim, Germany); (ii)metofluthrin (96%), prallethrin (93.3%) and pyriproxyfen (99.6%), from Sumitomo Chemical Co. Ltd. (Tokyo, Japan); and (iii) chlorfenapyr (100%) from BASF (Ludwigshafen, Germany). The initial stock solutions of each insecticide were prepared by diluting them in analytical grade acetone, with the exception of clothianidin and flupyradifurone whose stock solutions were prepared by dissolving them in a mixture of acetone and MERO® (0.903 density) according to the manufacturer’s instructions to prevent crystallization (Table 3). After a preliminary experiment to determine sublethal concentrations, MERO® was used at 1500 ppm (equivalent to 170 μl MERO® mixed in 100 ml of acetone) or 800 ppm (equivalent to 89 μl MERO® mixed in 100 ml of acetone) to prepare the stock solutions for coating bottles for testing *Aedes* spp. and *Anopheles* spp., respectively. For testing with *An. albimanus*, the concentration of MERO® was reduced to 200 ppm (equivalent to 22 μl MERO® mixed in 100 ml of acetone) to avoid high mortality in control mosquitoes. The glass bottles with stock solutions were then wrapped in aluminium foil to avoid exposure to UV radiation in sunlight and closed with tightly fitting caps to prevent evaporation of acetone before being stored at 4–6 °C until use. Ten-fold serial dilutions were then prepared from the stock solution.

**Table 3:** Optimised test conditions and specific endpoints for each insecticide and mosquito species in the WHO bottle assay

Insecticide class	Insecticide	Mosquito species	Bottle drying time (h)	Exposure time (h)	Recording time (h)	Surfactant and solvent control	Endpoint
Pyrroles	Chlorfenapyr	All *Anopheles* species	24	1	72	Acetone	Mortality
Neonicotinoids	Clothianidin	*An. gambiae, An. funestus, An. stephensi, An. minimus*	24	1	24	Acetone + MERO^a^ 800 ppm	
		*An. albimanus*	24	1	24	Acetone + MERO^a^ 200 ppm	
		*Ae. aegypti, Ae. albopictus*	24	1	24	Acetone + MERO^a^ 1500 ppm	
Butenolides	Flupyradifurone	*An. gambiae, An. funestus, An. stephensi, An. minimus*	24	1	24	Acetone + MERO^a^ 800 ppm	
		*An. albimanus*	24	1	24	Acetone + MERO^a^ 200 ppm	
		*Ae. aegypti, Ae. albopictus*	24	1	24	Acetone + MERO^a^ 1500 ppm	
Pyrethroids	Transfluthrin	All* Anopheles *and* Aedes *species	24	1	24	Acetone	
	Prallethrin	*Ae. aegypti, Ae. albopictus*	24	1	24	Acetone	
	Metofluthrin	*Ae. aegypti, Ae. albopictus*	24	1	24	Acetone	
Juvenile hormone mimics	Pyriproxyfen	*An. gambiae, An. funestus, An. stephensi*	2	1	72 h for mortality; 7 days for oviposition^b^	Acetone	Oviposition inhibition

*Ae. Aedes* mosquitoes,* An. Anopheles* mosquitoes

^a^MERO: 81% rapeseed oil methyl ester (Bayer CropScience)

^b^The 7-day period includes a 72-h holding period in which mosquitoes are kept in paper cups to record mortality, followed by an additional 96 h of individual chambering of surviving females to record oviposition

#### Process of coating and drying of bottles

In the testing laboratories, 250-ml Wheaton® bottles were coated according to the CDC guidelines [23]. Each bottle and its cap were coated with 1 ml of insecticide solution by rolling and inverting the bottle until all visible signs of liquid had disappeared. In parallel, a control bottle was coated with either 1 ml acetone alone or with 1 ml mixture of acetone and MERO® according to the solvent used for the insecticide solution. After coating, the bottles were opened and left horizontally in the dark to dry for 24 h at room temperature, except for pyriproxyfen-coated bottles that were dried for only 2 h before being used for testing.

#### Test conditions

To avoid any influence of environmental conditions on the test results, mosquitoes were maintained at 27 °C ± 2 °C and 80% ± 10% relative humidity during the exposure and holding periods.

#### Test procedures for insecticides with a killing action

The test conditions for bottle bioassays of each insecticide are summarized in Table 3, and the detailed standard operating procedure (SOP) is available from the WHO website [24]. Briefly, 100 non-blood-fed females, aged 3–5 days (4 replicates of 25 mosquitoes each), were exposed to a range of concentrations of the insecticide solution, obtained by serial dilutions, with at least five concentrations, for 1 h; two replicates of 25 mosquitoes were included as a control. After the exposure, mosquitoes were gently removed from the bottles using a mechanical aspirator, transferred into paper cups covered with netting and provided with cotton pads soaked in 10% sucrose solution. Knockdown was recorded at the end of the 1-h exposure period, and mortality was recorded at 24 h post exposure, except for the bottle bioassays with chlorfenapyr for which a 72-h holding period post exposure was found necessary for recording mortality in mosquitoes.

#### Test procedures for insecticides with sterilizing properties

Test conditions for pyriproxyfen, a juvenile hormone mimic with sterilizing properties, are summarized in Table 3, and the SOP is available from [25]. For pyriproxyfen, for which the outcome is OI, only 5- to 7-day-old, blood-fed female mosquitoes that were allowed to mate with healthy males for 2–3 days in colony cages prior to blood-feeding were used. The females were allowed to blood feed for 1 h prior to the test. Briefly, 100 female mosquitoes were exposed in four batches of 25 each for 1 h to each of the serial insecticide concentrations or a control. After exposure, mosquitoes were gently removed from the bottles using a mechanical aspirator, transferred into paper cups and provided access to cotton wool pads soaked in a 10% sucrose solution. Mortality was recorded up to 72 h after the initial 1-h exposure period. After 72 h, surviving females were individually kept in paper cups for another 96 h, and the proportion of females laying eggs and the number of eggs laid by each female were recorded for both the control and the treatment groups.

#### Endpoints for susceptibility testing

For insecticides with a killing action, the 24 h mortality, or 72 h mortality for chlorfenapyr, was used as the final endpoint for insecticide susceptibility. The mortality of the test sample or control was calculated from the sum of dead mosquitoes across replicates and expressed as a percentage of the total number of mosquitoes exposed. If the mortality in the control was ≥ 20%, the test was discarded and repeated. When control mortality was > 5% but < 20%, the test mortality was corrected with control mortality as part of the model fitting process, using methods analogous to the Abbott’s formula as per WHO guidelines [13]. For pyriproxyfen, the OI rate (OI%) was calculated as the proportion of egg-laying females exposed to pyriproxyfen against the proportion in the control, assessed at 7 days after the 1-h exposure. The total reduction in oviposition rate was obtained by calculating the percentage reduction in the number of females that laid eggs in treatments versus the number of females laying eggs in the control for each pyriproxyfen concentration. If the oviposition rate at the end of 7 days after exposure was < 30% in the control mosquitoes, the test was discarded and repeated.

### Statistical analysis

#### Fitting concentration–response relationships

A binomial model using a five-parameter logistic function was developed to analyse intensity bioassay data from the WHO multi-centre study. The same framework was used to analyse data from the killing bioassays and those parameters which affected the fecundity of mosquitoes. In both cases, a binomial sampling distribution was used to describe the outcome following exposure to the control or insecticide treatment:

$${y}_{i} \sim \mathrm{binomial}\left({n}_{i},{p}_{i}\right),$$

where ${y}_{i}$ is the number of mosquitoes that died or were inhibited from ovipositing in bioassay $i$; ${n}_{i}$ is the number of mosquitoes tested in the bioassay; and $0\le {p}_{i}\le 1$ is the mean proportion of mosquitoes that died or were inhibited from ovipositing, which is assumed to follow a logistic curve:

$${p}_{i}=D+ \frac{A-(D- Z)}{{[1+{e}^{B\cdot (\mathrm{ln}\left({x}_{i}\right)-C)}]}^{E}}.$$

Here, ${x}_{i}\ge 0$ is the insecticide concentration and* A*,* B*,* C*,* D* and* E* are all parameters that influence the shape and position of the logistic curve, while *A*, B,* D* and *E* are being strictly non-negative. For each unique intensity bioassay or set of bioassays run in each laboratory, and for each species and insecticide, the parameters were estimated by fitting the model to each data point within that group. In all runs, parameters *A* and *D* were set to 0 and 1, respectively, so that the resulting dose–response curve represents the estimated mortality without the background mortality, *Z*, with parameters *B*, *C*, *E* and *Z* varying by laboratory, species and insecticide.

The model was fitted using a Bayesian framework. Priors were defined as *B* ~ *N*(3,1), *C* ~ N(3,5), *E* ~ *N*(3,5) and* Z* ~ *N*(0,5). Individual parameter values for each curve can be found in Additional file 1: Table S1. The model was run using the probabilistic programming language Stan [26] in R v4.0.2 [27]. The model was run on four Markov chains for 5000 Markov Chain Monte Carlo (MCMC) iterations (or 10,000 iterations if the model did not converge at 5000), with 50% of iterations discarded as a warm-up. Non-convergence indicates that the MCMC sampler has not managed to sample from the true population distribution and may, therefore, bias the resulting estimates. Increasing the number of iterations while running the sampler enabled convergence to be reached more easily, but this procedure is computationally more expensive and so was only performed on those runs where convergence was not initially reached. The model was fitted to all bioassays from each laboratory for each unique combination of insecticide and species tested, generating one curve for each laboratory, insecticide and mosquito species. Uncertainty around this estimate was generated from the range of concentration–response curves provided by each bioassay. Within each laboratory, a curve was fitted to each individual bioassay replicate, and the minimum and maximum estimates around the point estimate were used to capture the uncertainty. A single curve indicates that there was only a single replicate for that combination of insecticide and mosquito species. Curves were plotted by extrapolating across the observed range of insecticide concentrations that were tested across all laboratories.

If multiple institutions provided data for specific insecticide and species combinations, the mean of the fitted estimates from these different institutions was computed when generating statistics at the insecticide and species level.

#### Model variability

Model variability was estimated by computing the absolute difference in mortality or OI of each individual data point from the best fit line for each MCMC iteration at the insecticide, species and location level. The median of all iterations for each insecticide, species and location was computed to generate a measure of variability, which is interpreted as the percentage variability in mortality or OI from the best fit line. This measure allows the amount of within-bioassay variability to be quantified in terms of mortality or OI. Estimates were generated for each mosquito species-insecticide combination, with the variability reflecting the average within-bioassay variability between different laboratories with its 95% confidence intervals.

Where multiple institutions provided data for specific insecticide and species combinations, the mean of the fitted estimates from these different institutions was computed when generating estimates for the insecticide and species levels.

## Results

### Baseline data

**Fig. 2:**
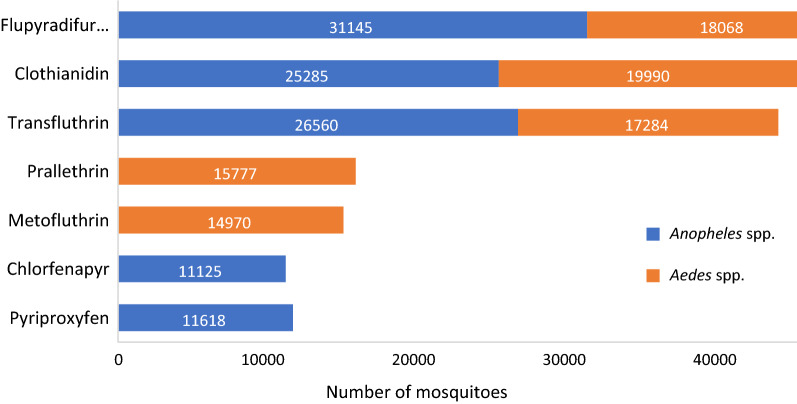
Total numbers of *Aedes* and *Anopheles* spp. used in WHO bottle assays according to the type of insecticide

Overall, 191,822 *Aedes* and *Anopheles* mosquitoes were used to test seven insecticides in the bottle bioassay (Fig. 2). About 5% of the mosquitoes (*n* = 10,345) were used for the method development and preliminary testing and thus were not included in the statistical analysis [22]. The other 181,477 mosquitoes were used to calculate the estimates for the concentration–responses plots (Table 4). The total number of mosquitoes tested per insecticide varied based on mosquito availability and testing capacity, and ranged from 440 for flupyradifurone in *An. funestus* s.s to 11,128 for clothianidin in *Ae. albopictus*, depending on the number of testing centres, number of concentrations tested and number of replicate bioassays needed. For each insecticide–species combination, a series of concentrations giving 0–100% mortality, or the OI for pyriproxyfen, were used to create concentration–response plots and estimate the LCs or OIs.

**Table 4:** Data used for the statistical analysis

Insecticide	Species	Number of data points	Mean number of mosquitoes per bioassay	Total number of mosquitoes analysed	Summary of mortality or oviposition inhibition (%)			Summary of concentrations (ug/bottle)		
					Minimum	Maximum	Mean	Minimum	Maximum	Mean
Clothianidin	*Ae. aegypti*	103	662	8602	0	100	56.4	0	20	1.5
	*Ae. albopictus*	128	795	11,128	0	100	50.7	0	10	1.4
Flupyradifurone	*Ae. aegypti*	111	552	8829	0	100	50.9	0	80	8.8
	*Ae. albopictus*	114	616	9239	0	100	63.8	0	80	9
Metofluthrin	*Ae. aegypti*	64	610	5491	0	100	50.2	0	1	0.2
	*Ae. albopictus*	107	622	9331	0	100	53.1	0	1	0.2
Prallethrin	*Ae. aegypti*	67	575	6326	0	100	49.3	0	30	6.1
	*Ae. albopictus*	109	630	9451	0	100	48.9	0	30	3.9
Transfluthrin	*Ae. aegypti*	104	537	8051	0	100	53.5	0	3	0.5
	*Ae. albopictus*	111	616	9233	0	100	53.8	0	3	0.4
Chlorfenapyr	*An. albimanus*	16	508	1015	6.6	100	58.7	0	200	39.7
	*An. funestus*	28	163	653	0	100	69.8	0	100	38.6
	*An. gambiae*	87	592	5332	0	100	58.5	0	200	28.8
	*An. stephensi*	50	426	2558	0	100	80.5	0	200	77.3
Clothianidin	*An. albimanus*	75	618	6183	0	100	57.5	0	6	0.8
	*An. funestus*	46	240	1917	0	100	65.1	0	20	1.6
	*An. gambiae*	77	669	6694	2	100	57.9	0	10	0.9
	*An. stephensi*	111	533	8532	0	100	42.3	0	10	0.9
Flupyradifurone	*An. albimanus*	50	668	4675	0	100	52.2	0	500	71.6
	*An. funestus*	11	220	440	0	100	48.5	0	100	25.5
	*An. gambiae*	98	630	7561	0	100	56.2	0	60	9.6
	*An. minimus*	81	644	7084	0	100	58.7	0	200	50.8
	*An. stephensi*	109	552	8273	0	100	55.5	0	60	10.5
Transfluthrin	*An. albimanus*	93	627	7527	0	100	49.6	0	2	0.3
	*An. funestus*	26	368	1470	0	100	51.1	0	2	0.4
	*An. gambiae*	92	496	6945	0	100	51.8	0	2	0.3
	*An. minimus*	28	436	1742	0	100	57.0	0	20	1.4
	*An. stephensi*	112	571	8561	0	100	52.8	0	2	0.2
Pyriproxyfen	*An. gambiae*	50	617	4317	0	100	75.0	0	800	80.8
	*An. stephensi*	58	480	4317	0	100	83.8	0	200	83.8

### Concentration–response curves

**Fig. 3:**
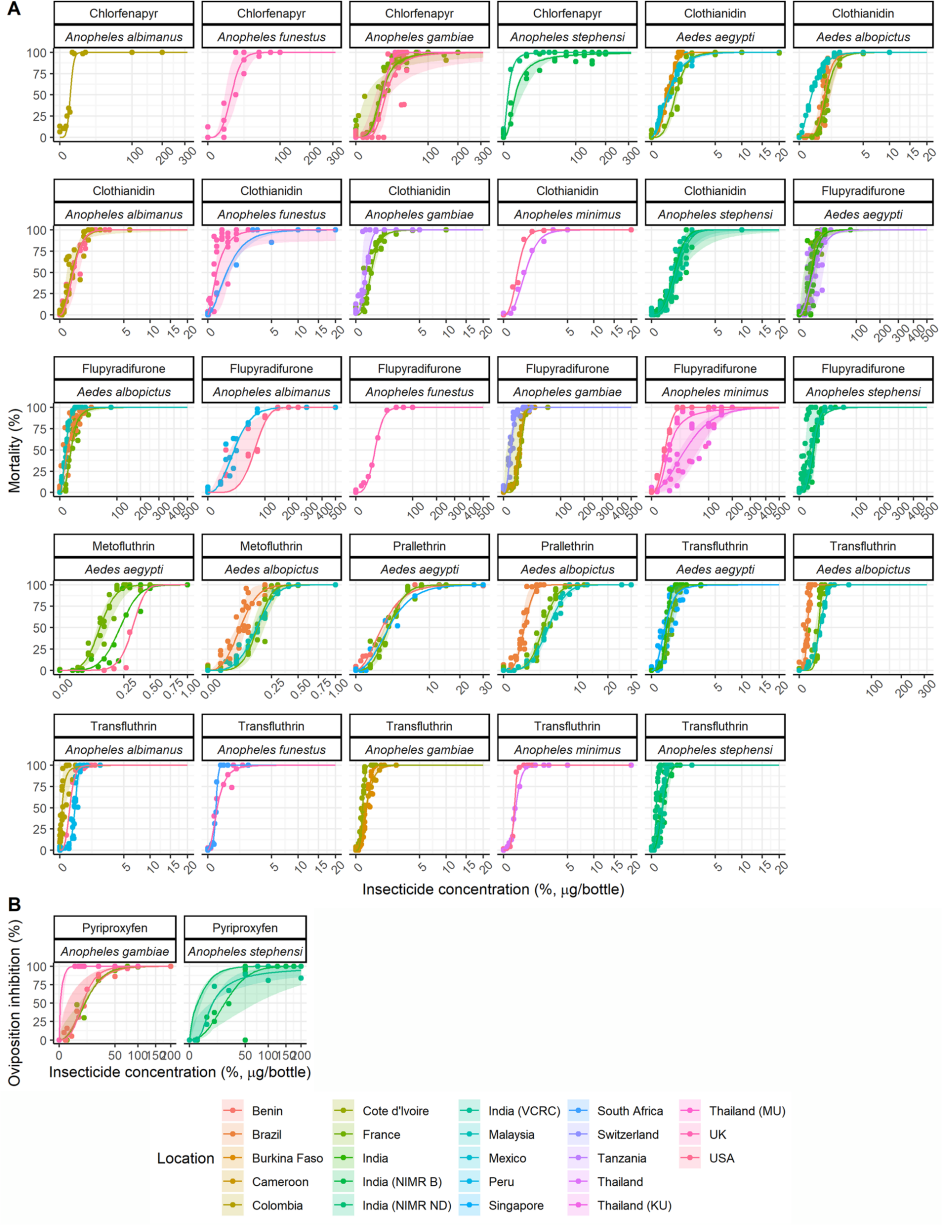
Concentration–response curves of each insecticide tested against *Aedes* and *Anopheles* species in WHO bottle bioassays at different test sites (coloured). **a** Insecticides for which percentage mortality was the primary endpoint, **b** insecticides for which oviposition inhibition rate (%) was the primary endpoint. Solid lines represent the posterior median concentration–response relationship of all fits for each test site, and the shaded area around the line represents the minimum and maximum bioassay estimates for that test site

The concentration–response curves estimated from the statistical analysis for each mosquito species and insecticide are shown in Fig. 3. For all species–insecticide combinations there are clear concentration–mortality trends (Fig. 3a). Large differences in LC_50_ and LC_99_ values were observed in different insecticides; for example higher LC estimate values were generated for flupyradifurone, chlorfenapyr and pyriproxyfen than for clothianidin and the pyrethroids (Table 5). Furthermore, LC_50_ estimates for the same insecticide were found to vary substantially between mosquito species; for example, the flupyradifurone LC_50_ was 5 (range 0.90–9.2) µg/bottle for *An. stephensi* and 44 (14–68) µg/bottle) for *An. albimanus.* The same was true with chlorfenapyr for which about a 10-fold difference in LC_50_ was observed between *An. stephensi* (1.5 [range 0.38–6.6] µg/bottle) and *An. gambiae* s.s (14 [2.2–21] µg/bottle)*.* For some other insecticides, only a little change in estimated LC_50_ values was observed between mosquito species; for example the LC_50_ for transfluthrin ranged from 0.09 (range 0.078–0.094) µg/bottle for *An. funestus* s.s to 0.15 (0.013–0.18) µg/bottle for *An. minimus* s.s. In addition, LC_50_ and LC_99_ values showed little variability between *Ae. aegypti* and *Ae. albopictus* for any insecticide (Table 5). The LC_50_ sometimes varied substantially between laboratories or countries for the same insecticide-species combination. For example, the *Ae. albopictus* LC_50_ for prallethrin varied from 0.78 (range 0.74–0.82) µg/bottle in Brazil to 3.7 (3.5–3.8) µg/bottle in Malaysia. All estimates of variability by institution are shown in Additional file 2: Table S2. In contrast, other mosquito–insecticide combinations were relatively consistent. For example, the estimated LC_50_ values for clothianidin against *An. stephensi* were 0.63 (range 0.59–0.67) µg/bottle at NIMR Bengaluru (India), 0.70 (0.66–0.75) µg/bottle at VCRC, Puducherry (India) and 0.80 (0.76–0.85 )µg/bottle at NIMR, New Delhi (India). The trend was similar for LC_99_ values used by the WHO to establish DCs (see Additional file 2: Table S2).

**Table 5:** Concentration–response statistics for all insecticides tested in WHO bottle assays and expected mortality or oviposition inhibition variability at the species level

Insecticide	Species	LC_50_ (median)	Range	LC_99_ (median)	Range	Variability of mortality (%)	95% Confidence interval	No. of locations
Clothianidin	*Ae. aegypti*	0.44	0.27–0.84	3.5	1.4–8.3	4.5	2.5–6.4	3
	*Ae. albopictus*	0.62	0.18–1.2	3.5	1.8–4.7	5.6	1.9–8.9	3
Flupyradifurone	*Ae. aegypti*	4.9	0.58–17.5	45	12–170	12	6.6–16	3
	*Ae. albopictus*	2.4	0.65–4.38	28	8–120	5.6	2.5–9.8	3
Metofluthrin	*Ae. aegypti*	0.21	0.08–0.32	0.49	0.26–0.59	6.9	4.9–10	3
	*Ae. albopictus*	0.12	0.049–0.19	0.37	0.14–0.60	5.8	2.8–10.0	3
Prallethrin	*Ae. aegypti*	1.7	1.5–2.2	13.5	6.4–19	4.0	2.6–5.1	3
	*Ae. albopictus*	2.4	0.5–3.9	7.6	1.6–13.7	4.8	2.9–6.4	3
Transfluthrin	*Ae. aegypti*	0.28	0.14–0.60	1.5	0.5–2.6	6.8	2.5–11	3
	*Ae. albopictus*	0.35	0.031–0.55	0.82	0.14–1.5	7.8	3.1–10	3
Chlorfenapyr	*An. albimanus*	1.9	1.9–2.0	6	5.9–6.0	6.6	5.9–7.5	1
	*An. funestus*	10	5.5–17	42	18–65	7.7	7.6–8.3	1
	*An. gambiae*	14	2.2–21	120	37–1.8e+04	8.5	3.0–13	4
	*An. stephensi*	1.5	0.38–6.6	200	16–820	3.4	1.3–5.1	2
Clothianidin	*An. albimanus*	0.19	0.055–0.45	1.8	0.88–46.8	6.9	4.1–9.6	2
	*An. funestus*	0.26	0.06–0.60	5.6	0.48–11.9	9.8	4.4–15	2
	*An. gambiae*	0.22	0.039–0.37	1.5	0.16–2.5	7.7	6.6–9.0	2
	*An. stephensi*	0.71	0.52–1.0	2.9	1.8–23.1	6.9	5.9–8.5	3
Flupyradifurone	*An. albimanus*	44	14–68	150	130–290	8.3	6.8–10	2
	*An. funestus*	11	11–11	43	43–43	2.8	1.9–4.9	1
	*An. gambiae*	5.9	1.0–9.3	17	6.5–22	4.4	2.1–6.4	3
	*An. minimus*	16	5.4–74	260	25–420	8.3	2.6–68	3
	*An. stephensi*	5.0	0.9–9.2	33	4–34	9.7	4.3–19	3
Transfluthrin	*An. albimanus*	0.13	0.003–0.30	0.60	0.017–1.1	7.3	2.0–13	3
	*An. funestus*	0.09	0.078–0.094	1.10	0.19–4.4	4.3	3.0–6.9	2
	*An. gambiae*	0.11	0.04–0.16	0.69	0.13–1.4	5.2	4.0–6.3	3
	*An. minimus*	0.15	0.013–0.18	0.58	0.31–0.83	1.6	1.1–2.6	2
	*An. stephensi*	0.10	0.025–0.27	0.44	0.095–1.2	6.5	3.5–8.1	3

Insecticide	Species	OI_50_ (median)	Range	OI_99_ (median)	Range	OI % variability	95% CI	No. of locations
Pyriproxyfen^a^	*An. gambiae*	7.6	0.25–10	62	1.5–110	2.5	6.6e−10–5.9	4
	*An. stephensi*	10	0.74–71	800	5.1–1.6e+05	3.4	6.1e−12–7.5	3

Model outputs at the insecticide-species-institution level were generated by computing the median of all model iterations. Summary statistics for each unique insecticide and species combination were generated from the mean of institution-level outputs (i.e. the median of all model iterations). The uncertainty around LC estimates was generated from the minimum and maximum (i.e. the range) bioassay-level estimates for each insecticide-species-institution combination. The mortality/OI variability was generated from the mean of institution-level estimates (generated as the median of all model iterations) per insecticide and species. The uncertainty around the variability estimate was provided by the 95% confidence intervals of the variability estimate: if multiple institutions contributed to the estimate for the insecticide-species combination, the minimum and maximum of these values were used to represent uncertainty; if only one institution informed the estimate, the actual 95% CIs around the median estimate are shown. The number of locations is the number of institutions for each combination (even if in the same country)

*LC*_*50*_, *LC*_*50*_ Lethal concentration that kills 50% and 90%, respectively, of the test population

^a^For pyriproxyfen, estimates were based on the 50% and 99% oviposition inhibition (OI_50_ and OI_99_, respectively)

The concentration–response curves for pyriproxyfen show broadly similar results although there are insufficient data to draw conclusion on the OI estimates for any species other than *An. gambiae* s.s. and *An. stephensi* (Fig. 3b). The average OI_50_ values did not vary much between these two species, varying from 7.6 (range 0.25–10) µg/bottle for *An. gambiae* to 10 (0.74–71) µg/bottle for *An. stephensi*. However, greater variability and difference between species were seen at the top of the curve, with an average OI_99_ of 62 (range 1.5–110) µg/bottle and 800 (5–1.6e+05) µg/bottle for *An. gambiae* s.s and *An. stephensi*, respectively. The raw data extracted from the experimental bioassays showed that the OI_100_, which was the lowest concentration inhibiting 100% oviposition in all bioassays, was 100 and 75 µg/bottle for *An. gambiae* and *An. stephensi*, respectively, with these values being more consistent.

### Variability in estimates

**Fig. 4:**
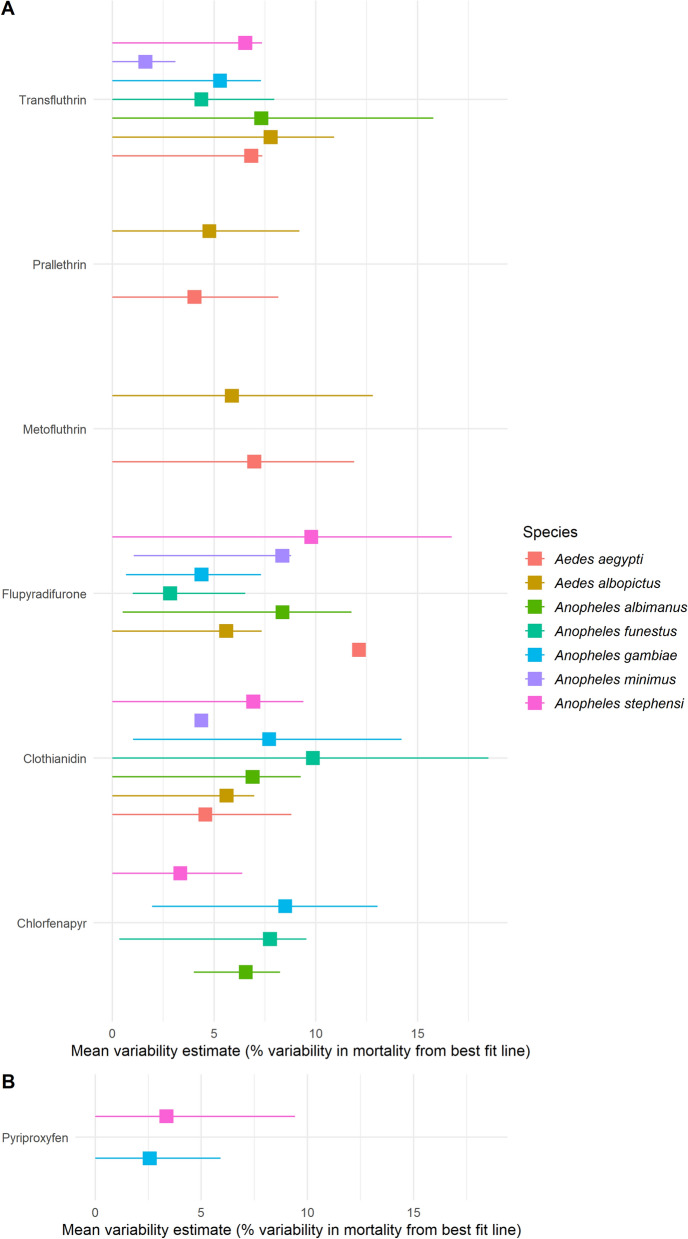
Estimated variability rates for mortality (**a**) and oviposition inhibition (**b**) for *Aedes* and *Anopheles* species in WHO bottle bioassays. The plots show the median variability estimate in either mortality or oviposition inhibition around the best fit line for each insecticide and species. Large squares represent the median estimate in variability of all model iterations for each mosquito species (as shown by colour) exposed to different insecticides. These points represent the within-bioassay variability across all data points. The error bars around that value represent the 95% confidence intervals. Values are interpreted as the percentage difference in within-bioassay variability in mortality or oviposition inhibition from the best fit line (median of all simulations)

The statistical model allows results from different concentration–response curves to be rigorously compared. The overall variability between the individual data points and the best fit mortality line is shown in Fig. 4. Our findings showed that the within-bioassay variability was relatively consistent across mosquito species for some insecticides, including chlorfenapyr and clothianidin. In contrast, for other insecticides, such as flupyradifurone or transfluthrin, it could vary substantially. Overall, the mean percentage variability in mortality from the best fit line was < 10% for all insecticides, except flupyradifurone for which variability in mortality was up to 12% for *Ae. aegypti* (Fig. 4). With pyriproxyfen, the mean within-assay variability in OI was 2.5% and 3.4% for *An. gambiae* s.s and *An. stephensi*, respectively.

## Discussion

This multi-centre study involving 21 laboratories worldwide was carried under the overall supervision of the WHO with the aim to develop and validate a bottle bioassay method by which to evaluate mosquito susceptibility and establish DCs for public health insecticides with physical properties that make them unsuitable for impregnation on filter papers. This bottle bioassay, modified from the US-CDC [19] protocol, was named the WHO bottle bioassay [22] and was calibrated to measure knockdown after 1 h of exposure and mortality after a holding period of 24 h (or up to 72 h for slow-acting compounds such as chlorfenapyr) to harmonize the susceptibility endpoints with those measured in the WHO tube test [13].

Overall, about 200,000 mosquitoes of two *Aedes* species (i.e. *Ae. aegypti* and *Ae. albopictus*) and five major malaria vectors in several regions (*An. gambiae* s.s, *An. funestus* s.s,* An. stephensi*, *An. minimus* s.s and *An. albimanus*) were tested with the WHO bottle bioassays, making this, to our knowledge, the largest mosquito toxicological dataset ever produced. Our findings showed that the WHO bottle bioassay was adequate to perform concentration–response analysis and calculate the toxicity (i.e. LC_50_/OI_50_ and LC_99_/OI_99_) of various insecticide class groups, namely the pyrethroids metofluthrin, prallethrin and transfluthrin; the neonicotinoid clothianidin; the pyrrole chlorfenapyr; the juvenile hormone mimic pyriproxyfen; and the butenolide flupyradifurone.

Despite the promising results obtained and some very consistent results, a marked variability in LC_50_ and LC_99_ estimates was seen for some mosquito species-insecticide combinations. The variability was generally higher with transfluthrin and flupyradifurone than with any other insecticide tested, especially against *Anopheles* species. For transfluthrin, which is a volatile pyrethroid, the loss of insecticide content in coated bottles during the 24-h drying time might explain the variation in the test results, especially when low concentrations were tested. The median LC_99_ ranged from 0.44 µg/bottle for *An. stephensi* to 1.5 µg/bottle for *Ae. aegypti* (Table 5). The use of MERO® as a surfactant to prevent the crystallization of flupyradifurone in bottles, in accordance with the manufacturer’s instructions, proved challenging, with some laboratories reporting high mortality rates (> 20%) in control mosquito batches, especially with *Anopheles* spp. Reducing the concentration of MERO® from 1500 to 800 ppm for all *Anopheles* spp. or to 200 ppm for *An. albimanus* was found necessary to reduce the mosquito mortality in the control bottles and improve consistency in the test results. With the exception of *An. funestus*, we did not encounter such issues with clothianidin despite the use of MERO® as a surfactant. The difference in the physico-chemical properties between butenolides and neonicotinoids together with the 2-log difference in the concentrations used for the bioassays (Table 4) might explain these outcomes.

With respect to the holding period after which mortality is scored in the WHO bottle bioassay, no increase in mortality was seen with clothianidin after 24–48 h or at 72 h post exposure, in contrast to other previous studies [16, 20]. Consequently, a holding period of 24 h was considered adequate for testing clothianidin. With chlorfenapyr, a pyrrole group compound, the bioassay results were more consistent when the temperature was not < 25 °C and the holding period was extended up to 72 h, in agreement with previous observations [17, 20].

Further test method optimization may however be needed, especially for volatile compounds that may evaporate more quickly than other insecticides, and for new classes of chemical compounds. For example, we could not develop adequate test protocols for three insecticides (dinotefuran, imidacloprid and indoxacarb) due to lack of consistency and repeatability within and between laboratories in bottle bioassays [22]. We assumed that the drying process might be critical for these three compounds and that bottle bioassays might also require the addition of a suitable surfactant to coat bottles adequately. Further work is necessary to develop test protocols for these compounds, which may be used increasingly in formulating vector control products.

Finally, the research of additional surfactants for the WHO bottle bioassays is of high priority considering that MERO® is currently produced by a single manufacturer (i.e. Bayer Crop Scienc, Leverkusen, Germnay). In addition, due to its oily composition, MERO® may facilitate insecticide uptake by increasing cuticular penetration [28], potentially underestimating resistance in wild mosquito populations tested using this method. Recent findings showed that SPAN 80, a non-ionic surfactant marketed by Sigma-Aldrich (Saint Louis, MO, USA), could be a good candidate for coating glass bottles with new chemical compounds such as neonicotinoids, and further investigations are ongoing to define the optimum concentrations of SPAN 80 to adopt for various chemicals and insect species (e.g. mosquitoes and sand flies).

In this study we also validated a new Bayesian binomial model using a 5-parameter logistic function developed at Imperial College London to analyse large and complex bioassay datasets. The model offers a flexible Bayesian alternative to common probit or log-probit analysis methods [29], allowing the integration of prior knowledge and generating novel ways to summarise the data. Overall, the model was shown to be adequate to produce concentration–response estimates and assess the variability in endpoints for different insecticides with particular modes of action. The model highlighted the high variability in estimates generated at different laboratories for some mosquito species–insecticide combinations and differences were observed in both the LCs and distribution of the LCs. There was an even greater uncertainty in the estimates for pyriproxyfen which has sterilizing properties compared to insecticides with a “killing” mode of action, probably due to the greater variability in control fecundity as compared to control mortality. With the Bayesian framework, uncertainty is integrated in the parameter estimates, generating posterior distributions rather than point estimates. To assess statistically significant difference between the different insecticide–strain combinations, further formal comparison tests could have been undertaken [30, 31]. Here, the range of estimated values from individual bioassays around LCs or OIs for each insecticide–species combination was provided to express uncertainty. Moreover, the small number of replicates generated for the same insecticide–species combination in different laboratories meant that the between-species and between-laboratory variability could not be determined. However, within-bioassay variability in susceptibility endpoints could be determined with the available data and was quantified by computing the absolute difference in mortality, or oviposition inhibition, of each data point from the best fit line at the insecticide, species, and location level. Our findings showed that the within-bioassay variability in either mortality or OI was < 10% for all insecticide–species combination, except for flupyradifurone for *Ae. aegypti*, indicating that the WHO bottle bioassay is a robust and reproducible method and can be advocated to evaluate the susceptibility of new public health insecticides.

Further work is ongoing to address the sources of variability (e.g. differences between mosquito strains or environmental conditions) as this information may allow the method parameters to be refined to help to reduce uncertainty and increase precision in estimates. Preliminary investigation showed that the variation or uncertainty in mortality is lower when LC_95_, LC_90_ or LC_80_ values are used rather than LC_99_ values, irregardless of the type of insecticide or species (see Additional file 3: Figure S1). The greater consistency when selecting lower LC values is due to the shape of the relationship and stochasticity. A better understanding of variability in mortality at the top of the concentration–response curves is deemed important, as this metric is used to determine insecticide DCs for routine resistance monitoring. More replicates from within the same laboratory could allow hierarchical models to be fit, allowing rigourous capturing of the within- and between-laboratory variability.

## Conclusion

This study provides strong evidence to support the use of the WHO bottle bioassay for assessing mosquito susceptibility to certain new public health insecticides that have the potential for use in vector control but that cannot be impregnated onto filter papers due to technical reasons. The method and the datasets presented in this study have been used recently to establish and validate 17 new WHO DCs for insecticides against either *Aedes* spp. or *Anopheles* spp. ([22]; Additional file 4: Table S3). The WHO bottle bioassay and the DCs determined in this study (see Additional file 4: Table S3) can now be widely used to monitor baseline susceptibility of wild populations of vectors of malaria and *Aedes-*borne diseases.

## Supplementary Information

**Additional file 1: Table S1.** Best fit model parameters for all insecticides–species combinations.

**Additional file 2: Table S2.** Concentration–response statistics and estimate variability for each insecticide–species combination at the institutional level.

**Additional file 3: Figure S1**. Variability in calculated lethal concentrations for some insecticide-species combinations (only chlorfenapyr, clothianidine and flupyradifurone are shown). The plots represent the uncertainty (density of estimates) of LC_80_, LC_90,_ LC_95_ and LC_99_ as shown by the Bayesian model. The larger the plot, the wider the variability of the estimate.

**Additional file 4: Table S3.** Insecticide discriminating concentrations for *Aedes *and *Anopheles* species in WHO bottle assays (source [22]).

## Data Availability

The datasets used and/or analysed during the current study are available from the corresponding author on reasonable request.

## Abbreviations

CDC: US Centers for Disease Control and Prevention
DC: Discriminating concentration
GVCR: Global Vector Control Response 2017–2030
LC: Lethal concentration
MCMC: Markov chain Monte Carlo
MERO®: 81% Rapeseed oil methyl ester
OI: Oviposition inhibition
SOP: Standard operating procedure
