# Supplementary material for: A new WHO bottle bioassay method to assess the susceptibility of mosquito vectors to public health insecticides: results from a WHO-coordinated multi-centre study
Source: Parasit Vectors. 2023 Jan 20;16:21. doi: 10.1186/s13071-022-05554-7 (PMC9863080; doi:10.1186/s13071-022-05554-7)
Supplement: Supplementary file 1 — Additional file 1: Table S1. Best fit model parameters for all insecticides–species combinations. [file 13071_2022_5554_MOESM1_ESM.docx]

**Additional file 1:** Table S1. Best fit model parameters for all insecticides-species combinations.

| **Insecticide** | **Species** | **Country** | **Institution** | **B** | **C** | **E** | **Z** |
| --- | --- | --- | --- | --- | --- | --- | --- |
| Clothianidin | *Aedes aegypti* | Burkina Faso | IRSS | 2.633 | 0.442 | 10.9 | 0.056 |
| Clothianidin | *Aedes aegypti* | France | IRD | 3.463 | 0.138 | 2.394 | 0.034 |
| Clothianidin | *Aedes aegypti* | Mexico | UANL | 1.577 | 0.823 | 7.268 | 0.004 |
| Clothianidin | *Aedes albopictus* | Brazil | FIOCRUZ | 6.087 | -0.103 | 1.148 | 0.007 |
| Clothianidin | *Aedes albopictus* | France | IRD | 5.676 | -0.044 | 1.059 | 0.008 |
| Clothianidin | *Aedes albopictus* | Malaysia | VCRU | 1.545 | 0.69 | 8.384 | 0.005 |
| Flupyradifurone | *Aedes aegypti* | France | IRD | 5.252 | 0.629 | 0.76 | 0.017 |
| Flupyradifurone | *Aedes aegypti* | India | NIMR ND | 2.133 | 2.004 | 10.7 | 0.017 |
| Flupyradifurone | *Aedes aegypti* | Tanzania | LSHTM-KCMC | 1.807 | 2.289 | 9.207 | 0.051 |
| Flupyradifurone | *Aedes albopictus* | Brazil | FIOCRUZ | 1.397 | 2.152 | 10.6 | 0.077 |
| Flupyradifurone | *Aedes albopictus* | France | IRD | 3.063 | 0.824 | 1.61 | 0.012 |
| Flupyradifurone | *Aedes albopictus* | Malaysia | VCRU | 1.857 | 1.417 | 11.1 | 0.011 |
| Metofluthrin | *Aedes aegypti* | France | IRD | 3.992 | -0.741 | 4.205 | 0.005 |
| Metofluthrin | *Aedes aegypti* | India | NIMR ND | 4.853 | -0.345 | 5.931 | 0.006 |
| Metofluthrin | *Aedes aegypti* | USA | CDC | 8.281 | -0.257 | 9.677 | 0.008 |
| Metofluthrin | *Aedes albopictus* | Brazil | FIOCRUZ | 2.751 | -0.423 | 10.1 | 0.048 |
| Metofluthrin | *Aedes albopictus* | France | IRD | 5.082 | -0.455 | 9.18 | 0.033 |
| Metofluthrin | *Aedes albopictus* | Malaysia | VCRU | 6.677 | -0.205 | 11.8 | 0.011 |
| Prallethrin | *Aedes aegypti* | Benin | LSHTM-CREC | 1.93 | 1.226 | 6.581 | 0.042 |
| Prallethrin | *Aedes aegypti* | France | IRD | 2.883 | 1.189 | 8.872 | 0.015 |
| Prallethrin | *Aedes aegypti* | Singapore | NEA | 2.14 | 1.279 | 6.086 | 0.008 |
| Prallethrin | *Aedes albopictus* | Brazil | FIOCRUZ | 3.552 | 0.518 | 7.237 | 0.024 |
| Prallethrin | *Aedes albopictus* | France | IRD | 4.226 | 1.021 | 6.207 | 0.019 |
| Prallethrin | *Aedes albopictus* | Malaysia | VCRU | 6.643 | 1.385 | 10.9 | 0.004 |
| Transfluthrin | *Aedes aegypti* | France | IRD | 3.748 | -0.058 | 4.131 | 0.01 |
| Transfluthrin | *Aedes aegypti* | India | NIMR ND | 5.037 | -0.189 | 7.764 | 0.0006 |
| Transfluthrin | *Aedes aegypti* | Singapore | NEA | 2.446 | -0.295 | 2.779 | 0.022 |
| Transfluthrin | *Aedes albopictus* | Brazil | FIOCRUZ | 4.887 | -0.904 | 4.67 | 0.101 |
| Transfluthrin | *Aedes albopictus* | France | IRD | 9.175 | -0.279 | 2.606 | 0.064 |
| Transfluthrin | *Aedes albopictus* | Malaysia | VCRU | 4.197 | 0.338 | 11.8 | 0.03 |
| Chlorfenapyr | *Anopheles albimanus* | Colombia | NIH C | 5.084 | 0.587 | 2.745 | 0.093 |
| Chlorfenapyr | *Anopheles funestus* | UK | LSTM | 3.656 | 1.637 | 4.64 | 0.053 |
| Chlorfenapyr | *Anopheles gambiae* | Cote d'Ivoire | LSHTM-IPR | 5.212 | 1.212 | 0.774 | 0.13 |
| Chlorfenapyr | *Anopheles gambiae* | France | IRD | 4.44 | 1.173 | 0.819 | 0.133 |
| Chlorfenapyr | *Anopheles gambiae* | UK | LSTM | 4.468 | 1.78 | 6.668 | 0.024 |
| Chlorfenapyr | *Anopheles gambiae* | USA | CDC | 6.184 | 1.289 | 0.585 | 0.044 |
| Chlorfenapyr | *Anopheles stephensi* | India | NIMR B | 2.775 | 0.249 | 0.678 | 0.049 |
| Chlorfenapyr | *Anopheles stephensi* | India | NIMR ND | 1.667 | 0.614 | 4.507 | 0.055 |
| Clothianidin | *Anopheles albimanus* | Colombia | NIH C | 1.743 | 0.496 | 8.678 | 0.027 |
| Clothianidin | *Anopheles albimanus* | USA | CDC | 2.053 | 0.595 | 12.1 | 0.045 |
| Clothianidin | *Anopheles funestus* | South Africa | NICD | 1.687 | 0.683 | 5.259 | 0.022 |
| Clothianidin | *Anopheles funestus* | UK | LSTM | 1.334 | 0.508 | 7.37.046 | 0.05 |
| Clothianidin | *Anopheles gambiae* | France | IRD | 3.306 | -0.441 | 1.68 | 0.063 |
| Clothianidin | *Anopheles gambiae* | Tanzania | LSHTM-KCMC | 2.651 | -0.08 | 9.744 | 0.101 |
| Clothianidin | *Anopheles stephensi* | India | NIMR B | 3.048 | 0.599 | 9.351 | 0.015 |
| Clothianidin | *Anopheles stephensi* | India | NIMR ND | 3.093 | 0.631 | 7.76 | 0.005 |
| Clothianidin | *Anopheles stephensi* | India | VCRC | 3.056 | 0.584 | 7.962 | 0.058 |
| Flupyradifurone | *Anopheles albimanus* | Peru | NIH P | 2.337 | 2.531 | 8.195 | 0.033 |
| Flupyradifurone | *Anopheles albimanus* | USA | CDC | 4.909 | 2.654 | 11 | 0.151 |
| Flupyradifurone | *Anopheles funestus* | UK | LSTM | 3.411 | 1.852 | 6.902 | 0.036 |
| Flupyradifurone | *Anopheles gambiae* | Cameroon | OCEAC | 5.854 | 1.216 | 2.76 | 0.029 |
| Flupyradifurone | *Anopheles gambiae* | Cote d'Ivoire | LSHTM-IPR | 5.521 | 1.552 | 9.292 | 0.034 |
| Flupyradifurone | *Anopheles gambiae* | Switzerland | Swiss TPH | 2.648 | 0.628 | 0.884 | 0.064 |
| Flupyradifurone | *Anopheles minimus* | Thailand | KU | 1.855 | 3.112 | 9.173 | 0.023 |
| Flupyradifurone | *Anopheles minimus* | Thailand | MU | 3.562 | 0.912 | 0.642 | 0.017 |
| Flupyradifurone | *Anopheles minimus* | USA | CDC | 2.673 | 1.868 | 11 | 0.063 |
| Flupyradifurone | *Anopheles stephensi* | India | NIMR B | 3.191 | 1.772 | 11.1 | 0.045 |
| Flupyradifurone | *Anopheles stephensi* | India | NIMR ND | 1.546 | 2.268 | 9.894 | 0.053 |
| Flupyradifurone | *Anopheles stephensi* | India | VCRC | 3.088 | 1.49 | 5.772 | 0.011 |
| Transfluthrin | *Anopheles albimanus* | Colombia | NIH C | 1.424 | -2.073 | 1.965 | 0.07 |
| Transfluthrin | *Anopheles albimanus* | Peru | NIH P | 5.66 | -0.247 | 7.87 | 0.014 |
| Transfluthrin | *Anopheles albimanus* | USA | CDC | 3.415 | -0.723 | 2.958 | 0.061 |
| Transfluthrin | *Anopheles funestus* | South Africa | NICD | 4.792 | -0.878 | 5.125 | 0.017 |
| Transfluthrin | *Anopheles funestus* | UK | LSTM | 1.97 | -0.258 | 4.639 | 0.029 |
| Transfluthrin | *Anopheles gambiae* | Burkina Faso | IRSS | 3.136 | -0.14 | 9.993 | 0.015 |
| Transfluthrin | *Anopheles gambiae* | Cameroon | OCEAC | 1.923 | 0.096 | 7.122 | 0.03 |
| Transfluthrin | *Anopheles gambiae* | Cote d'Ivoire | LSHTM-IPR | 4.382 | -0.941 | 5.78 | 0.033 |
| Transfluthrin | *Anopheles minimus* | Thailand | KU | 3.16 | -0.281 | 4.9 | 0.01 |
| Transfluthrin | *Anopheles minimus* | USA | CDC | 5.567 | -0.651 | 5.18 | 0.009 |
| Transfluthrin | *Anopheles stephensi* | India | NIMR B | 2.62 | -0.035 | 8.464 | 0.011 |
| Transfluthrin | *Anopheles stephensi* | India | NIMR ND | 3.615 | -1.359 | 4.12 | 0.011 |
| Transfluthrin | *Anopheles stephensi* | India | VCRC | 4.072 | -0.286 | 11.6 | 0.022 |
| Pyriproxyfen | *Anopheles gambiae* | Benin | LSHTM-CREC | 2.013 | 2.201 | 6.341 | 0.005 |
| Pyriproxyfen | *Anopheles gambiae* | UK | LSTM | 2.683 | -1.353 | 3.298 | 0.008 |
| Pyriproxyfen | *Anopheles gambiae* | France | IRD | 2.251 | 2.172 | 7.304 | 0.006 |
| Pyriproxyfen | *Anopheles gambiae* | USA | CDC | 3.14 | 1.528 | 3.337 | 0.005 |
| Pyriproxyfen | *Anopheles stephensi* | India | NIMR B | 2.294 | 2.745 | 12.7 | 0.002 |
| Pyriproxyfen | *Anopheles stephensi* | India | NIMR ND | 2.795 | 0.425 | 4.285 | 0.008 |
| Pyriproxyfen | *Anopheles stephensi* | India | VCRC | 4.632 | 0.62 | 0.316 | 0.005 |
