# Supplementary material for: A new WHO bottle bioassay method to assess the susceptibility of mosquito vectors to public health insecticides: results from a WHO-coordinated multi-centre study
Source: Parasit Vectors. 2023 Jan 20;16:21. doi: 10.1186/s13071-022-05554-7 (PMC9863080; doi:10.1186/s13071-022-05554-7)
Supplement: Supplementary file 2 — Additional file 2: Table S2. Concentration–response statistics and estimate variability for each insecticide–species combination at the institutional level. [file 13071_2022_5554_MOESM2_ESM.docx]

**Additional file 2** Table S2. Concentration-response statistics and estimate variability for each insecticide-species combination at the institutional level.

| Insecticide | Species | Country | Institution | LC_50_ (mean) | 95% CIs | LC_99_  (mean) | 95% CIs | Mortality / OI variability | 95% CIs |
| --- | --- | --- | --- | --- | --- | --- | --- | --- | --- |
| Clothianidin | *Aedes aegypti* | Burkina Faso | IRSS | 0.313 | 0.281-0.351 | 1.549 | 1.362-1.81 | 5.083 | 4.858-5.947 |
| Clothianidin | *Aedes aegypti* | France | IRD | 0.708 | 0.661-0.757 | 3.847 | 3.215-4.83 | 5.968 | 5.765-6.375 |
| Clothianidin | *Aedes aegypti* | Mexico | UANL | 0.308 | 0.279-0.338 | 5.22 | 4.258-7.052 | 2.656 | 2.503-3.092 |
| Clothianidin | *Aedes albopictus* | Brazil | FIOCRUZ | 0.77 | 0.744-0.796 | 3.166 | 2.607-4.042 | 8.58 | 8.401-8.905 |
| Clothianidin | *Aedes albopictus* | France | IRD | 0.894 | 0.865-0.926 | 4.374 | 3.572-5.546 | 6.145 | 5.949-6.34 |
| Clothianidin | *Aedes albopictus* | Malaysia | VCRU | 0.18 | 0.161-0.20 | 3.033 | 2.493-3.959 | 2.103 | 1.983-2.456 |
| Flupyradifurone | *Aedes aegypti* | France | IRD | 4.118 | 3.92-4.324 | 37.4 | 28.5-51.2 | 6.866 | 6.663-7.212 |
| Flupyradifurone | *Aedes aegypti* | India | NIMR ND | 4.517 | 4.149-4.922 | 32.5 | 27.4-40.1 | 13.8 | 13.5-14.2 |
| Flupyradifurone | *Aedes aegypti* | Tanzania | LSHTM-KCMC | 6.159 | 5.605-6.778 | 66.6 | 54.6-87.74 | 15.7 | 15.5-16.2 |
| Flupyradifurone | *Aedes albopictus* | Brazil | FIOCRUZ | 1.645 | 1.288-2.055 | 33.8 | 28.1-42.3 | 9.066 | 8.679-9.782 |
| Flupyradifurone | *Aedes albopictus* | France | IRD | 3.631 | 3.318-3.962 | 42.8 | 29.9-68.6 | 5.047 | 4.879-5.407 |
| Flupyradifurone | *Aedes albopictus* | Malaysia | VCRU | 0.916 | 0.83-1.025 | 8.774 | 7.52-10.4 | 2.685 | 2.749-3.1 |
| Metofluthrin | *Aedes aegypti* | France | IRD | 0.098 | 0.093-0.102 | 0.336 | 0.309-0.373 | 5.165 | 5.028-5.363 |
| Metofluthrin | *Aedes aegypti* | India | NIMR ND | 0.222 | 0.206-0.237 | 0.584 | 0.51-0.727 | 9.589 | 8.861-10.6 |
| Metofluthrin | *Aedes aegypti* | USA | CDC | 0.323 | 0.307-0.34 | 0.54 | 0.494-0.607 | 6.175 | 4.895-7.785 |
| Metofluthrin | *Aedes albopictus* | Brazil | FIOCRUZ | 0.064 | 0.058-0.07 | 0.3 | 0.26-0.358 | 9.317 | 8.983-10 |
| Metofluthrin | *Aedes albopictus* | France | IRD | 0.15 | 0.143-0.157 | 0.349 | 0.329-0.374 | 5.326 | 5.19-5.642 |
| Metofluthrin | *Aedes albopictus* | Malaysia | VCRU | 0.146 | 0.139-0.155 | 0.453 | 0.42-0.493 | 2.992 | 2.82-3.34 |
| Prallethrin | *Aedes aegypti* | Benin | LSHTM-CREC | 1.257 | 1.014-1.562 | 13.7 | 10.3-19.5 | 2.946 | 2.616-3.742 |
| Prallethrin | *Aedes aegypti* | France | IRD | 1.947 | 1.838-2.064 | 8.665 | 7.884-.683 | 4.502 | 4.248-4.954 |
| Prallethrin | *Aedes aegypti* | Singapore | NEA | 1.999 | 1.66-2.367 | 18.2 | 13.6-30.4 | 4.658 | 4.485-5.09 |
| Prallethrin | *Aedes albopictus* | Brazil | FIOCRUZ | 0.784 | 0.745-0.825 | 2.718 | 2.463-3.076 | 5.635 | 5.411-6.454 |
| Prallethrin | *Aedes albopictus* | France | IRD | 2.889 | 2.767-3.017 | 8.554 | 7.918-9.461 | 5.6 | 5.47-5.83 |
| Prallethrin | *Aedes albopictus* | Malaysia | VCRU | 3.652 | 3.482-3.823 | 11.6 | 10.7-12.7 | 3.072 | 2.94-3.26 |
| Transfluthrin | *Aedes aegypti* | France | IRD | 0.37 | 0.352-0.388 | 1.399 | 1.268-1.622 | 6.148 | 5.938-6.457 |
| Transfluthrin | *Aedes aegypti* | India | NIMR ND | 0.271 | 0.26-0.283 | 0.648 | 0.598-0.714 | 2.695 | 2.52-3.134 |
| Transfluthrin | *Aedes aegypti* | Singapore | NEA | 0.218 | 0.199-0.24 | 2.538 | 1.83-4.263 | 11.6 | 11.5-11.9 |
| Transfluthrin | *Aedes albopictus* | Brazil | FIOCRUZ | 0.077 | 0.072-0.082 | 0.205 | 0.191-0.224 | 10.1 | 9.97-10.4 |
| Transfluthrin | *Aedes albopictus* | France | IRD | 0.445 | 0.434-0.456 | 0.826 | 0.781-0.888 | 9.856 | 9.396-10.4 |
| Transfluthrin | *Aedes albopictus* | Malaysia | VCRU | 0.524 | 0.494-0.553 | 1.413 | 1.312-1.546 | 3.357 | 3.123-3.821 |
| Chlorfenapyr | *Anopheles albimanus* | Colombia | NIH C | 1.987 | 1.775-2.223 | 5.985 | 4.965-8.099 | 6.559 | 5.946-7.508 |
| Chlorfenapyr | *Anopheles funestus* | UK | LSTM | 10.4 | 8.804-12.1 | 42.3 | 33.8-59.9 | 7.749 | 7.683-8.263 |
| Chlorfenapyr | *Anopheles gambiae* | Cote d'Ivoire | LSHTM-IPR | 13.2 | 11.5-14.9 | 1.20E+02 | 77.6-2.40E+02 | 10.4 | 9.304-11.7 |
| Chlorfenapyr | *Anopheles gambiae* | France | IRD | 12 | 10.7-13.5 | 1.60E+02 | 1.1E+02-2.6E+02 | 7.272 | 6.697-8.566 |
| Chlorfenapyr | *Anopheles gambiae* | UK | LSTM | 13.4 | 11.9-15.1 | 37.5 | 32.8-44.6 | 3.044 | 3.044-4.404 |
| Chlorfenapyr | *Anopheles gambiae* | USA | CDC | 17.3 | 16-18.6 | 1.80E+02 | 1.3E+02-2.6E+02 | 12.6 | 12-13.4 |
| Chlorfenapyr | *Anopheles stephensi* | India | NIMR ND | 2.595 | 1.935-3.555 | 3.90E+02 | 2.2E+02-7.7E+02 | 4.895 | 4.717-5.132 |
| Chlorfenapyr | *Anopheles stephensi* | India | NIMR B | 0.348 | 0.077-0.743 | 15.1 | 7.365-40.5 | 1.776 | 1.338-2.677 |
| Clothianidin | *Anopheles albimanus* | Colombia | NIH C | 0.167 | 0.15-0.186 | 2.011 | 1.685-2.574 | 9.445 | 9.299-9.638 |
| Clothianidin | *Anopheles albimanus* | USA | CDC | 0.213 | 0.182-0.246 | 1.609 | 1.149-1.873 | 4.336 | 4.116-5.122 |
| Clothianidin | *Anopheles funestus* | South Africa | NICD | 0.428 | 0.282-0.618 | 8.327 | 5.73-15.2 | 4.926 | 4.427-5.838 |
| Clothianidin | *Anopheles funestus* | UK | LSTM | 0.096 | 0.074-0.122 | 2.848 | 1.988-5.008 | 14.8 | 14-15.8 |
| Clothianidin | *Anopheles gambiae* | France | IRD | 0.282 | 0.262-0.304 | 2.399 | 1.829-3.374 | 6.872 | 6.67-7.244 |
| Clothianidin | *Anopheles gambiae* | Tanzania | LSHTM-KCMC | 0.122 | 0.109-0.137 | 0.613 | 0.522-0.738 | 8.546 | 8.249-9.009 |
| Clothianidin | *Anopheles stephensi* | India | NIMR B | 0.636 | 0.598-0.675 | 2.593 | 2.332-2.975 | 6.594 | 6.422-6.898 |
| Clothianidin | *Anopheles stephensi* | India | NIMR ND | 0.804 | 0.763-0.847 | 3.355 | 2.905-4.251 | 5.993 | 5.93-6.19 |
| Clothianidin | *Anopheles stephensi* | India | VCRC | 0.703 | 0.66-0.749 | 2.977 | 2.618-3.644 | 8.198 | 8.069-8.499 |
| Flupyradifurone | *Anopheles albimanus* | Peru | NIH P | 21 | 19.2-23 | 1.40E+02 | 1.1E+02-1.8E+02 | 6.848 | 6.798-7.185 |
| Flupyradifurone | *Anopheles albimanus* | USA | CDC | 67 | 62.8-70.8 | 1.60E+02 | 1.4E+02-1.7E+02 | 9.871 | 9.33-10.4 |
| Flupyradifurone | *Anopheles funestus* | South Africa | NICD | 11.3 | 8.953-14.1 | 43.7 | 31.3-68.9 | 2.84 | 1.889-4.927 |
| Flupyradifurone | *Anopheles gambiae* | Cameroon | OCEAC | 70.505 | 7.234-7.789 | 19.6 | 17.7-22.2 | 4.914 | 4.665-5.327 |
| Flupyradifurone | *Anopheles gambiae* | Cote d'Ivoire | LSHTM-IPR | 8.966 | 8.603-9.332 | 19.5 | 18.3-21.1 | 2.239 | 2.095-2.691 |
| Flupyradifurone | *Anopheles gambiae* | Switzerland | Swiss TPH | 1.372 | 1.235-1.517 | 12.1 | 9.773-16.3 | 5.897 | 5.806-6.431 |
| Flupyradifurone | *Anopheles minimus* | Thailand | KU | 34.5 | 30.9-38.6 | 3.50E+02 | 3.0E+02-4.4E+02 | 9.925 | 9.84-10.1 |
| Flupyradifurone | *Anopheles minimus* | Thailand | MU | 9.123 | 8.164-10.2 | 4.00E+02 | 2.6E+02-6.7E+02 | 11.7 | 11.3-12.1 |
| Flupyradifurone | *Anopheles minimus* | USA | CDC | 5.492 | 4.672-6.442 | 26.7 | 22.5-32.5 | 3.471 | 2.892-4.705 |
| Flupyradifurone | *Anopheles stephensi* | India | NIMR B | 6.317 | 5.816-6.843 | 23.5 | 21.7-25.9 | 5.779 | 5.35-6.217 |
| Flupyradifurone | *Anopheles stephensi* | India | NIMR ND | 3.264 | 2.747-3.932 | 52 | 36.8-77.1 | 19 | 18.9-19.4 |
| Flupyradifurone | *Anopheles stephensi* | India | VCRC | 5.499 | 5.169-5.836 | 25.2 | 22.2-30.8 | 4.529 | 4.369-4.78 |
| Transfluthrin | *Anopheles albimanus* | Colombia | NIH C | 0.005 | 0.004-0.006 | 0.687 | 0.325-2.099 | 12.6 | 12.4-13.1 |
| Transfluthrin | *Anopheles albimanus* | Peru | NIH P | 0.267 | 0.259-0.275 | 0.58 | 0.547-0.622 | 6.656 | 6.548-6.852 |
| Transfluthrin | *Anopheles albimanus* | USA | CDC | 0.114 | 0.087-0.144 | 0.671 | 0.507-0.998 | 2.752 | 2.054-3.983 |
| Transfluthrin | *Anopheles funestus* | South Africa | NICD | 0.077 | 0.071-0.085 | 0.207 | 0.175-0.262 | 3.342 | 3.057-4.274 |
| Transfluthrin | *Anopheles funestus* | UK | LSTM | 0.107 | 0.072-0.147 | 2.065 | 1.346-7.777 | 5.404 | 4.537-6.992 |
| Transfluthrin | *Anopheles gambiae* | Burkina Faso | IRSS | 0.145 | 0.137-0.154 | 0.56 | 0.517-0.619 | 5.969 | 5.791-6.322 |
| Transfluthrin | *Anopheles gambiae* | Cameroon | OCEAC | 0.124 | 0.101-0.151 | 1.335 | 0.994-2.124 | 4.659 | 4.093-5.555 |
| Transfluthrin | *Anopheles gambiae* | Cote d'Ivoire | LSHTM-IPR | 0.06 | 0.057-0.063 | 0.169 | 0.154-0.19 | 5.247 | 5.159-5.414 |
| Transfluthrin | *Anopheles minimus* | Thailand | KU | 0.178 | 0.152-0.209 | 0.832 | 0.624-1.262 | 1.578 | 1.138-2.616 |
| Transfluthrin | *Anopheles minimus* | USA | CDC | 0.137 | 0.124-0.151 | 0.32 | 0.28-0.382 | 1.661 | 1.228-2.45 |
| Transfluthrin | *Anopheles stephensi* | India | NIMR B | 0.149 | 0.139-0.159 | 0.786 | 0.693-0.921 | 7.71 | 7.447-7.989 |
| Transfluthrin | *Anopheles stephensi* | India | NIMR ND | 0.027 | 0.025-0.029 | 0.109 | 0.097-0.14 | 4.025 | 3.545-4.674 |
| Transfluthrin | *Anopheles stephensi* | India | VCRC | 0.145 | 0.138-0.152 | 0.403 | 0.38-0.435 | 7.867 | 7.644-8.101 |
| Pyriproxyfen | *Anopheles gambiae* | France | IRD | 10.9 | 9.234-12.7 | 81.5 | 65.9-1.1E+02 | 4.117 | 3.998-4.593 |
| Pyriproxyfen | *Anopheles gambiae* | USA | CDC | 8.987 | 7.762-10.1 | 66.4 | 51.6-94.1 | 1.028 | 0.655-1.666 |
| Pyriproxyfen | *Anopheles gambiae* | Benin | LSHTM-CREC | 10.4 | 8.823-11.7 | 1.00E+02 | 76.8-1.3E+02 | 4.796 | 4.58-5.069 |
| Pyriproxyfen | *Anopheles gambiae* | UK | LSTM | 0.023 | 4.2e-04-0.885 | 0.29 | 0.009-3.788 | 3.30E-05 | 6.6E-10-0.124 |
| Pyriproxyfen | *Anopheles stephensi* | India | NIMR B | 20.1 | 18-22.2 | 1.20E+02 | 1.1E+02-1.3E+02 | 3.354 | 3.038-3.714 |
| Pyriproxyfen | *Anopheles stephensi* | India | NIMR ND | 1.263 | 0.001-22 | 9.675 | 0.021-88.3 | 2.70E-05 | 6.1E-12-0.222 |
| Pyriproxyfen | *Anopheles stephensi* | India | VCRC | 8.665 | 7.121-10.3 | 2.30E+03 | 1.0E+03-5.4E+03 | 6.955 | 6.543-7.469 |
