# Supplementary material for: A new WHO bottle bioassay method to assess the susceptibility of mosquito vectors to public health insecticides: results from a WHO-coordinated multi-centre study
Source: Parasit Vectors. 2023 Jan 20;16:21. doi: 10.1186/s13071-022-05554-7 (PMC9863080; doi:10.1186/s13071-022-05554-7)

**Additional file 3. Figure S1**. Variability in calculated Lethal Concentrations for some insecticide-species combinations (only chlorfenapyr, clothianidine and flupyradifurone were shown). The plots represent the uncertainty (density of estimates) of LC_80_, LC_90,_ LC_95_ and LC_99_ as shown by the Bayesian model. The larger the plot, the wider the variability of the estimate.


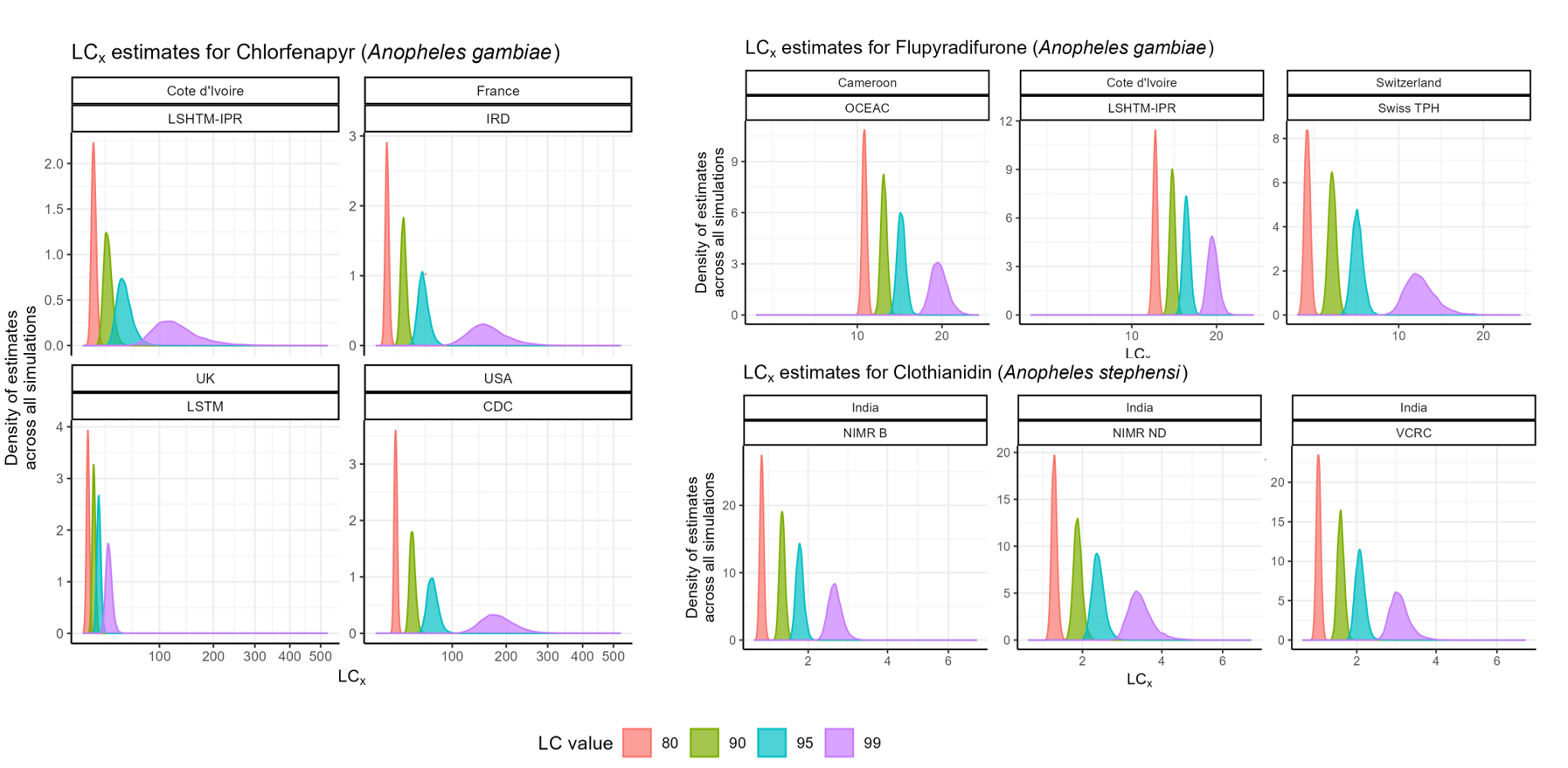

Supplement: Supplementary file 3 — Additional file 3: Figure S1. Variability in calculated lethal concentrations for some insecticide-species combinations (only chlorfenapyr, clothianidine and flupyradifurone are shown). The plots represent the uncertainty (density of estimates) of LC80, LC90, LC95 and LC99 as shown by the Bayesian model. The larger the plot, the wider the variability of the estimate. [file 13071_2022_5554_MOESM3_ESM.docx]
