# Supplementary material for: A new WHO bottle bioassay method to assess the susceptibility of mosquito vectors to public health insecticides: results from a WHO-coordinated multi-centre study
Source: Parasit Vectors. 2023 Jan 20;16:21. doi: 10.1186/s13071-022-05554-7 (PMC9863080; doi:10.1186/s13071-022-05554-7)
Supplement: Supplementary file 4 — Additional file 4: Table S3. Insecticide discriminating concentrations for Aedes and Anopheles species in WHO bottle assays (source [22]). [file 13071_2022_5554_MOESM4_ESM.docx]

**Additional file 4.** Table S3. Insecticide discriminating concentrations for *Aedes* and *Anopheles* species in WHO bottle assays (source [22])

| **Insecticide class** | **Insecticide** | **Targeted mosquito species** | **Discriminating concentration^a^** | **Exposure period (hour)** | **Holding period (hour)** | **Carrier oil / solvent/ surfactant** |
| --- | --- | --- | --- | --- | --- | --- |
| Pyrethroids | Transfluthrin | *An. albimanus*, *An. stephensi*, *An. funestus*, *An. minimus*, *An. gambiae* | 2 µg/bottle | 1 h | 24 h | Acetone only |
|  |  | *Ae. aegypti*, *Ae. albopictus* | 3 µg/bottle | 1 h | 24 h | Acetone only |
|  | Metofluthrin | *Ae. aegypti*, *Ae. albopictus* | 1 µg/bottle | 1 h | 24 h | Acetone only |
|  | Prallethrin | *Ae. aegypti*, *Ae. albopictus* | 30 µg/bottle | 1 h | 24 h | Acetone only |
| Neonicotinoids | Clothianidin | *An. albimanus, An. stephensi* | 10 µg/bottle | 1 h | 24 h | Acetone + MERO 800 ppm^c^ |
|  |  | *An. funestus s.s., An. gambiae s.s.* | 4 µg/bottle | 1 h | 24 h | Acetone + MERO 800 ppm^c^ |
|  |  | *An. minimus* | 6 µg/bottle | 1 h | 24 h | Acetone + MERO 800 ppm^c^ |
|  |  | *Ae. aegypti* | 20 µg/bottle | 1 h | 24 h | Acetone + MERO 1500 ppm^c^ |
|  |  | *Ae. albopictus* | 10 µg/bottle | 1 h | 24 h | Acetone + MERO 1500 ppm^c^ |
| Butenolides | Flupyradifurone | *An. albimanus* | 500 µg/bottle | 1 h | 24 h | Acetone + MERO 200 ppm^c^ |
|  |  | *An. stephensi, An. gambiae* s.s. | 60 µg/bottle | 1 h | 24 h | Acetone + MERO 200 ppm^c^ |
|  |  | *An. funestus* s.s., *An. minimus* | 100 µg/bottle | 1 h | 24 h | Acetone + MERO 200 ppm^c^ |
|  |  | *Ae. aegypti, Ae. albopictus* | 80 µg/bottle | 1 h | 24 h | Acetone + MERO 1500 ppm^c^ |
| Pyrroles | Chlorfenapyr | *An. gambiae* s.s., *An. stephensi*, *An. funestus* s.s., *An. albimanus* | 100 µg/bottle | 1 h | 72 h | Acetone only |
| JH hormone mimics | Pyriproxyfen | *An. gambiae* s.s., *An. stephensi*, *An. funestus* s.s. | 100 µg/bottle | 1 h | 72 h for mortality, 7 days^b^ for oviposition inhibition | Acetone only |

^a^ Bottle bioassay: DC in µg/bottle (250 mL).

^b^ The 7-day period includes a 72-h holding period in which mosquitoes are kept in paper cups to record mortality, followed by an additional 96 h of individual chambering of surviving females to record oviposition

^c^ MERO: 81% rapeseed oil methyl ester (manufactured by Bayer CropScience).
